# Rapidly expanding thenar eminence ganglion: a case report

**DOI:** 10.1186/1757-1626-2-129

**Published:** 2009-02-06

**Authors:** EA Azzopardi, S Gujral, A Mandal, M Kulkarni

**Affiliations:** 1 Specialty Registrar, Department of Plastic Reconstructive and Aesthetic Surgery, Wexham Park Hospital, Slough, UK

## Abstract

**Introduction:**

This study documents the first reported case of a rapidly growing (volar) thenar eminence ganglion arising form the first carpometacarpal joint, masquerading as a sarcoma. The discussion informs the hand surgeon on the evidence regarding the unusual presenting features.

**Case presentation:**

An 85 year old left hand dominant female presented with a six week history of rapidly growing lump on the thenar eminence. Clinical examination revealed a non-tender large lobulated mobile swelling measuring 5 × 4 cm and involving the whole thenar eminence.

**Conclusion:**

Ganglia may present from the thenar eminence and are a source of diagnostic confusion.

## Introduction

A rapidly expanding (volar) thenar eminence ganglion is a rare entity that may easily be confused with malignant soft tissue tumors. To our knowledge this the first reported case in the literature, both in site of (thenar eminence) and its unusually rapid expansion (5 × 4 cm in 6 weeks).

## Case presentation

**Figure 1:**
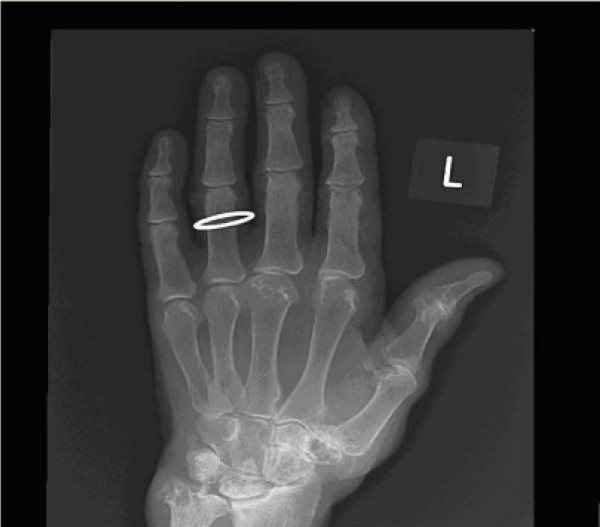
**Plain x-ray appearance of the degenerative changes in the first carpometacarpal joint**.

**Figure 2:**
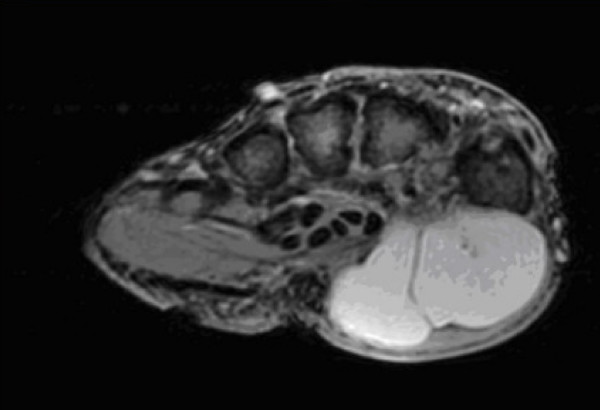
**MRI appearance of thenar eminence ganglion**.

An 85 year old left hand dominant female presented with a six week history of rapidly growing lump on the thenar eminence. Clinical examination revealed a non-tender large lobulated mobile swelling measuring 5 × 4 cm and involving the whole thenar eminence. A sarcoma was initially suspected. Plain X-ray (figure 1) revealed severe osteopenia with erosive changes in the first Carpometacarpal joint, and a soft tissue swelling around an osteophyte detached from the base of the first metacarpal. MRI reported a septate thin walled cystic structure extending from the first carpo-metacarpal joint space against a background of diffuse active synovitis (figure 2). In operating theater, a multilobulated lesion with a neck extending to the first CMC joint was observed. The histological analysis revealed a 3.4 g previously opened specimen consistent with a ganglion cyst. The patient made an excellent post-operative recovery with no evidence of recurrence.

## Discussion

In an effort to increase the rigor of our study by reducing retrieval bias, the structured search is being reported. This was performed using the search-construct "((Thenar OR Palmar OR Volar) AND Ganglion)" across the following databases: Pubmed EMBASE, AMED, CINAHL.

While dorsal and wrist ganglia are common in the upper limb, our literature search retrieved only one other article describing a volar wrist ganglion. Our case is materially different since Chiu and Aschermann reported a slowly enlarging ganglion arising from the wrist whereas the case reported here derived from the first carpometacarpal joint, expanded volarly, and rapid expanded over six weeks to the reported dimensions. Chiu and Aschermann (1993) reported an 'extensive review of the literature' to claim the first report of a ganglion presenting as a thenar mass. However they did not report a reproducible literature e search strategy to substantiate their claim.

Our patient presented at an atypical 85 years of age, in an atypical volar position and in an atypical rate of growth. Such a presentation may lead the unsuspecting clinician to disregard the possibility of this diagnosis when examining a thenar eminence lump. Conversely although soft tissue sarcomas of the hand are uncommon, the most frequent presentation is a painless soft tissue mass in the thenar eminence. The clinical confusion with sarcomas may prevent diagnostic aspiration, due to fears about seeding the tumor. This distinction between the two pathological entities is paramount due to the differences between operative resection, and the distinctly worse prognosis for palmar sarcomas compared to similar lesions elsewhere in an extremity.

## Conclusion

Ganglia may present from the thenar eminence and are a source of diagnostic confusion. Documentation of an occurrence of thenar ganglion may alert the hand surgeon to consider its possibility in planning diagnostic and operative procedures, and avoid patients' undue anxiety.

## Competing interests

The authors declare that they have no competing interests.

## Authors' contributions

AM, and SG contributed towards retrieval of data. EA performed the literature meta-search and was a major contributor in writing the manuscript. MK was essential for delivering a clinical perspective and critically reviewing the manuscript.

## Consent

Written informed consent was obtained from the patient for publication of this case report and accompanying images. A copy of the written consent is available for review by the Editor-in-Chief of this journal.
